# Mr. Bryer, on a Case of Bronchotomy

**Published:** 1800-03

**Authors:** Stephen P. Bryer

**Affiliations:** Surgeon and Agent for Sick and Wounded Seamen and Marines at the Port of Weymouth


### *To the Editors of the Medical and Physical Journal.*

GENTLEMEN, *Weymouth, Jan.* 16, 1800.

IF you perceive any thing new, or practically useful, in the following Case, it is at your service, and I should be much gratified by its insertion in your valuable publication.

I was sent for, about eight o'clock in the morning of the 14th of November last, to visit a woman in the Island of Portland, who, as the messenger informed me, had cut her throat in so dangerous a manner, that her friends did not imagine she could possibly survive till my arrival. I found her in convulsions, and apparently expiring; her pulse imperceptible, and her extremities quite cold. I found a gaping transverse wound, of about seven inches in extent, with very ragged edges; it having been inflicted with an old rusty knife, nearly in the shape of a pruning knife. The trachea was most completely divided about an inch below the larynx, quite through to the œsophagus, which was also slightly wounded. By her exertions, the divided extremities of the trachea were separated from each other full an inch. The carotid arteries, particularly the left, were laid bare, but fortunately only very slightly scratched. The left jugular vein was entirely divided, and had bled a considerable quantity; but the person who first went to her assistance, (almost at the moment of the accident) seeing the blood pouring from so large an orifice, with great presence of mind, instantly placed a plug of wet rag on the vessel, and kept his thumb thumb firm on it, till I arrived, which was about three hours after the wound was inflicted. I firft took up the vein, which bled very profufely on the preffure being removed; and after carefully removing as much as poffible of the coagulated blood, which had plugged up the extremities of the trachea, I united the integuments by about half a dozen ftitches, taking care to include in them as much as I conveniently could of the adipofe fubftance and mufcular fibres of the trachea. By confining her head to her breaft with the greateft attention, and permitting no other food, during the firft week, than that afforded by nutritive injection, fhe happily recovered, without any unpleafant fymptom whatever; except that, on the third day, on attempting to fwallow a little thin gruel, a great part of it made its way through the wound, and loofened the adhefive plaifter which I had applied to fupport the ftitches.

The patient was about fixty years of age, of a regular and temperate habit, and the mother of a large family. I am,

Gentlemen,

Your moft obedient humble fervant,

STEPHEN P. BRYER,

Surgeon and Agent for Sick and Wounded Seamen and Marines at the Port of Weymouth.

